# Correction to “Severe acute respiratory syndrome coronavirus 2 and preterm birth, clinical implications—A register‐based cohort study from Sweden”

**DOI:** 10.1002/pmf2.70206

**Published:** 2026-01-12

Berglin, L, K. Källén, B. Jacobsson, V. Sengpiel. “Severe Acute Respiratory Syndrome Coronavirus 2 and Preterm Birth, Clinical Implications—A Register‐Based Cohort Study From Sweden.” *Pregnancy* 1(6): e70136.

Following publication, the authors became aware that the data presented in the paper were based on the initial linkage between the Pregnancy Register and the SmiNet (Swedish Register for Surveillance of Communicable Diseases) including only a random sample of 25% of all individuals with a positive SARS‐CoV‐2 test which was used when testing the models. After re‐analysis in the total population, the overall results and odds ratios presented of the original paper remained essentially unchanged, but the absolute numbers in the original paper did not accurately reflect the full population. The authors have now corrected all numerical errors, using the full data set, including replacement of all figures.

We apologize for this error.

